# Lack of clear national policy guidance on COVID-19 vaccines influences behaviors in pregnant and lactating women in Kenya

**DOI:** 10.1080/21645515.2022.2127561

**Published:** 2022-10-31

**Authors:** Eleonor Zavala, Berhaun Fesshaye, Clarice Lee, Stephen Mutwiwa, Wincate Njagi, Paul Munyao, Rosemary Njogu, Rachel Gur-Arie, Alicia M. Paul, Taylor A. Holroyd, Prachi Singh, Ruth A. Karron, Rupali J. Limaye

**Affiliations:** a Department of International Health, Johns Hopkins University, Bloomberg School of Public Health, Baltimore, MD, USA; b Jhpiego, Nairobi, Kenya; c Berman Institute of Bioethics, Johns Hopkins University, Bloomberg School of Public Health, Baltimore, MD, USA; d International Vaccine Access Center, Johns Hopkins University, Bloomberg School of Public Health, Baltimore, MD, USA; e Department of Health, Behavior, and Society, Johns Hopkins University, Bloomberg School of Public Health, Baltimore, MD, USA; f Center for Immunization Research, Department of Epidemiology, Johns Hopkins University, Bloomberg School of Public Health, Baltimore, MD, USA; g Department of Epidemiology, Johns Hopkins University, Bloomberg School of Public Health, Baltimore, MD, USA

**Keywords:** Maternal immunization, COVID-19 vaccine, health policy, vaccine behavior, qualitative, Kenya

## Abstract

SARS-CoV-2 infection in pregnancy is associated with a greater risk of maternal and newborn morbidity and maternal death. In Kenya, pregnant and lactating women (PLW) were ineligible to receive COVID-19 vaccines until August 2021. How shifts in policy influence vaccine behaviors, such as health worker recommendations and vaccine uptake, is not well documented. We conducted qualitative interviews with PLW, health workers, and policymakers in Kenya to understand how different stakeholders’ perceptions of national policy regarding COVID-19 vaccination in pregnancy shaped vaccine behaviors and decision-making. Policymakers and health workers described pervasive uncertainty and lack of communication about the national policy, cited vaccine safety as their primary concern for administering COVID-19 vaccines to PLW, and expressed that PLW were inadequately prioritized in the COVID-19 vaccine program. PLW perceived the restrictive policy as indicative of a safety risk, resulting in vaccine hesitancy and potentially exacerbated inequities in vaccine access. These findings support the need for the development and dissemination of effective vaccine communication guidelines and the prioritization of PLW in COVID-19 vaccination policies and campaigns. To ensure PLW do not face the same inequities in future epidemics, data on infectious disease burdens and vaccine uptake should be collected systematically among pregnant women, and PLW should be included in future vaccine trials.

## Introduction

The first COVID-19 vaccines arrived in Kenya in March 2021, one year after the country’s first reported case.^1,2^ Frontline health and critical service workers were the first groups eligible for vaccination, followed by the elderly and those with comorbidities.^3^ Missing among the vaccine deployment and prioritization plans were pregnant and lactating women (PLW), who were explicitly excluded from vaccine eligibility. Like Kenya, several countries declared PLW ineligible for vaccine administration in the early months of vaccine delivery and roll-out, citing a lack of vaccine safety data in these populations.^4^

Studies indicated that pregnant women with COVID-19 had a greater risk of hospitalization, admission to the ICU, need for ventilation and oxygenation, and death compared to non-pregnant women with COVID-19.^5–9^ Pregnant women who contracted SARS-CoV-2 were more likely to experience preterm birth, neonatal intensive care unit (NICU) admission, and death compared to those who did not contract the virus.^5,7^ These findings led countries and international bodies to issue guidance recommending COVID-19 vaccine use in pregnancy, particularly in regions with adequate vaccine supply.^4^ In Kenya, PLW remained ineligible for vaccine administration until August 2021, when the Ministry of Health issued a statement permitting the use of a COVID-19 vaccine in a pregnant or lactating person only after a risk-benefit assessment and discussion with a provider.^10^

Recent post-authorization evidence indicates that COVID-19 vaccines in pregnancy do not increase the chances of miscarriage, preterm birth, or other adverse events.^11–16^ Compared to non-vaccinated pregnant individuals, vaccinated pregnant women are less likely to contract SARS-CoV-2, experience severe illness, or be admitted to critical care.^17–20^ Studies have found that COVID-19 vaccines during pregnancy confer protective antibodies to newborns,^21^ resulting in fewer hospitalizations of young infants born to vaccinated mothers compared to unvaccinated.^22^ In December 2021, the Kenya Director General of Health issued a letter to county health directors recommending COVID-19 vaccines in pregnancy with no additional requirements or qualifications.^23^

Despite evidence and recommendations, uptake of COVID-19 vaccines is suboptimal in many countries. As of 31 July 2022, approximately 9.3 million adults (34%) in Kenya were fully vaccinated for COVID-19.^24^ There are no data on COVID-19 vaccine uptake for PLW in Kenya but studies in other countries have found systematically lower rates among pregnant women compared to the general population.^25^ While vaccine supply was limited in the early months of the vaccine roll-out, Kenya had received 32 million doses as of May 2022, enough to cover roughly 32.7% of the total population, or 58.7% of the adult population, with a full course regimen.^26^

Vaccine policy is an important cornerstone for access to vaccines, including eligibility. Vaccine policy provides a framework for recommendations and delivery, and as such, can directly influence vaccine behaviors. This study investigated how policymakers, health workers, and PLW perceptions of COVID-19 vaccine policy influenced vaccine behaviors among PLW in Kenya.

## Materials and methods

### Participants and study area

**Figure 1.**
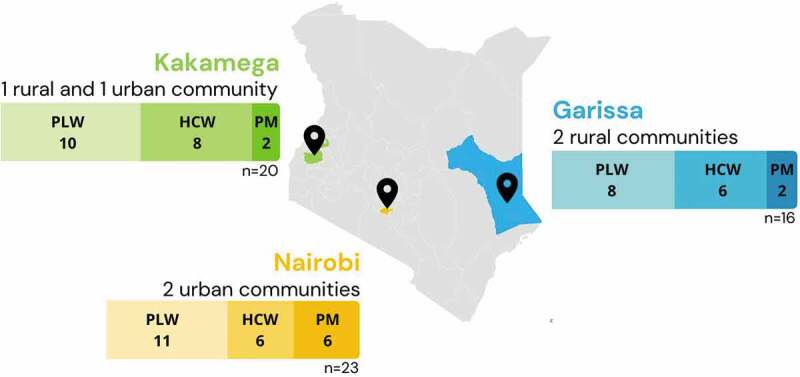
Study population and setting. Study area for sampling included two rural communities in Garissa county, two urban communities in Nairobi county, and one rural and one urban community in Kakamega county. PLW = pregnant and lactating women; HCW = health care workers; PM = policymakers.

In-depth interviews (IDIs) were conducted with 29 PLW, 20 health workers, and 10 policymakers for a total of 59 IDIs. Participants were recruited from Garissa, Kakamega, and Nairobi counties, across three urban communities and three rural communities to try and obtain a representative sample across the country (Figure 1). PLW and health workers were identified through health centers in each community, and health workers were selected based on whether they provided care to PLW, such as midwives, OB/GYNs, and family medicine providers. Policymakers were selected based on their professional involvement in immunization policy and programming and/or maternal health.

### Data collection

Data were collected from August 8^th^ to September 3^rd^, 2021. Semi-structured interview guides included questions related to the COVID-19 vaccine decision-making process for PLW and the current policy recommendations (see Supplementary Material). A three-day data collection training was held, consisting of field ethics, interview techniques, and pre-testing. Participants were recruited from health clinics for PLW and health workers, and via email/phone for policymakers. After determining eligibility and interest, oral informed consent was obtained. IDIs were conducted in either English, Swahili, or a local language in semi-private settings or via Zoom. Interviews were audio recorded, transcribed, and translated into English. All data were stored on encrypted servers.

### Contextual considerations

Prior to the start of data collection, PLW were considered ineligible for vaccination, per the national vaccination plan.^3^ On August 13^th^ 2021, the Kenya Obstetrical and Gynecological Society issued a statement recommending COVID-19 vaccines for PLW, citing the increased risks associated with COVID-19 during pregnancy and the post-authorization evidence on vaccine safety in pregnancy.^27^ The following week, the Ministry of Health released a press statement clarifying that PLW could choose to be vaccinated after receiving counseling on the benefits and risks.^10^ On December 24^th^, 2021, the Ministry of Health issued updated guidance stating that pregnant women should be offered mRNA vaccines.^23^

### Data analysis

Seven team members analyzed the data using a grounded theory approach, which uses an inductive approach to identify emerging themes and sub-themes. Following three rounds of open coding, a code list was developed (see Supplementary Material). Inter-rater reliability was conducted by two team members using 10% of the transcripts with reliability of >90%. Themes and sub-themes were agreed upon with all members. Data management was conducted with Atlas.ti software. This study received ethical approval from the Kenya Medical Research Institute and the Institutional Review Board of the Johns Hopkins Bloomberg School of Public Health (IRB00014893).

## Results

Four major themes emerged from participants’ perceptions of policy for the use of COVID-19 vaccines for PLW. Participants expressed uncertainty regarding the eligibility of PLW, described a lack of communication and guidance of the national policy for vaccine use in PLW, identified safety as the primary consideration for recommendations for PLW, and highlighted the need for PLW prioritization in policy planning.

### Uncertainty about COVID-19 vaccine eligibility for PLW

Overall, participants described uncertainty about the eligibility of PLW in Kenya related to COVID-19 vaccines. Some policymakers described the policy as prohibitive for the administration of COVID-19 vaccines to PLW, whereas others detailed the guidance as being more permissive, highlighting the requirement of an individual risk assessment before administration. The majority of health workers reported that PLW were not eligible for vaccination and described how this policy led to PLW not presenting at vaccination sites or being turned away. PLW described hesitancy in receiving the vaccine because of their perceived ineligibility, including those PLW who, due to their occupation as health care worker or teacher, belonged to a priority group for vaccination.

When asked if there was any policy guidance for the use of vaccines in pregnancy and lactation, some policymakers expressed uncertainty: “Policy by the government? That one I am not very sure about” (Policymaker, Nairobi, urban); and “Is there a current policy in place on COVID-19 vaccination in pregnant and lactating mothers? I am not sure. It’s not there?” (Policymaker, Garissa, rural). One policymaker said there was no policy: “We don’t have a specific policy on pregnant mothers as far as COVID-19 vaccine is concerned.” (Policymaker, Nairobi, urban).

In contrast, descriptions of eligibility and prioritization for other high-risk groups were clearly articulated: “In this country the priority people were: number one, healthcare workers, number two, the teachers and the armed soldiers and then the elderly” (Policymaker, Nairobi, urban). Whether PLW belonging to those prioritization groups were eligible to receive a vaccine was ambiguous, as described by this policymaker: “I think when you said health workers and when you say the vaccine, everybody was a target group but nobody said specifically pregnant mothers that are health workers” (Policymaker, Garissa, rural). A policymaker from Nairobi stated that pregnant women who were also health care workers were eligible: “Yeah, they are being prioritized. You know the health care providers are given priority … whether they are pregnant or not” (Policymaker, Nairobi, urban). Conversely, another described the exclusion of PLW within health worker prioritization: “They [pregnant and lactating health workers] were not included because the vaccine was not being given to lactating mothers and pregnant mothers so they didn’t get” (Policymaker, Kakamega, urban). A school teacher highlighted how the differing guidance for teachers and for pregnant women impacted her decision-making: “On the side of the teacher they said they should be vaccinated then on the other hand you hear pregnant women are not getting it … If not for this pregnancy, I could have gotten it” (Lactating mother, Nairobi, urban).

Many policymakers and health workers explained that PLW were specifically excluded from COVID-19 vaccination per government directives: “We don’t vaccinate in the first trimester and we also wait until after 6 months (post delivery) … It is the communication from the Ministry of Health” (Policymaker, Kakamega, urban). One policymaker detailed how this policy of exclusion was implemented at vaccination centers through a screening process: “She will be asked if she is pregnant. In fact, that is the first question that they are asked. In screening before you are given the jab: pregnancy, last menstrual period, this is one of the questions they will ask … the staff has to ascertain that you are not pregnant and you are not breastfeeding and then you are given a jab” (Policymaker, Kakamega, urban). At the policy implementation level, the majority of health workers expressed that they were not vaccinating pregnant women, citing vaccination policy: “As per the protocol we were given, the pregnant mothers should not get the vaccine. Yeah so we just encourage them to take precautions” (Health worker, Kakamega, urban). One health worker described how PLW were aware of the policy, and therefore did not seek out vaccination: “They are just screened out and they don’t get the vaccine. In fact most of them … they have already gotten the information that pregnant and breastfeeding mothers should not be vaccinated” (Health worker, Kakamega, urban). Pregnant women described not wanting the vaccine because they were told they were not eligible: “I was told that pregnant mothers are not being given the vaccine so that is what I have heard about it so I have never thought of having it” (Pregnant woman, Garissa, rural). Another woman explained how she was told to get the vaccine only after delivery. When asked if she had received counseling on why she was not eligible, she responded: “They did not tell us; we were just told not to go for the vaccines and wait after delivery, that is when we can go for it” (Pregnant woman, Nairobi, urban). Another expressed disappointment when told they were ineligible: “When they told us that we could not get the vaccine because we are pregnant, we thought, what could we do” (Pregnant woman, Nairobi, urban).

Some policymakers said the guidance for administering vaccines in PLW was not outrightly prohibitive and cited Ministry of Health guidance issued in mid-August 2021 that established an individual risk assessment requirement before vaccination. As one policymaker explained, “Currently, they [PLW] are only given [the vaccine] when benefits outweigh the risks, that means every case has to be assessed and if that person who is assessing feels that they’ll benefit, they’ll receive the vaccine once their risk profile has been assessed” (Policymaker, Nairobi, urban). Another policy maker from Nairobi referred to this assessment: “It’s our precaution so you weigh the benefits versus the risk. So I cannot give a blanket (that PLW) are not receiving. We are advising the health workers to weigh the benefits versus the risk, if the risk outweighs the benefit and they are counseled, then they can receive the vaccine” (Policymaker, Nairobi, urban). Despite this more permissive guidance, one policymaker acknowledged that the “benefits versus risks” policy generally resulted in the exclusion of pregnant women: “For pregnant women, currently the policy is that they are only given the vaccine if the benefits outweigh the risk, however giving in pregnancy is a precaution and therefore in most situations you will find that pregnant people are not given the vaccine unless they are at a high risk of COVID-19” (Policymaker, Nairobi, urban).

### Unclear communication related to COVID-19 vaccine policy for PLW

Most policymakers and health workers stressed the need for better communication of the national policy for COVID-19 vaccine use in PLW. The lack of clear guidance was identified as a major factor that affected PLWs’ vaccine decision-making.

Policymakers identified a need for explicit communication of the government’s policy of COVID-19 vaccine administration in pregnancy and lactation: “They [PLW] had been excluded before, but currently they are not, but the information is not adequate. Many people will still have questions in their mind of whether they are eligible for the vaccines and they need to be reassured of their eligibility” (Policymaker, Nairobi, urban). Another described pregnant women as being scared to take the vaccine, calling for communication to reduce hesitancy: “If [the vaccine] is not advocated for, you may not take it there and you will not vaccinate yourself. Because they are scared. They are scared of covid [vaccine]. Unless they are assured it will be useful for them and their babies, they are not likely to turn up. So there is a need for consistent advocacy and correct information” (Policymaker, Nairobi, urban). One policymaker pointed out that while the Kenya Obstetrical and Gynecological Society had issued recommendations for PLW, clear policy guidance from the government was still required for health workers to implement recommendations: “For example, we have the Kenya Obstetrical and Gynecological Society, they have produced guidelines on access of the vaccines to the mothers and it is available on their website. I am not sure how many in the general community are able to access their website … Then in terms of giving this information to the public we leave it to the government to be principled in terms of reassuring people that vaccines are safe for them. And once that policy pronouncement has been made by the government then the rest of us from the health care sector, especially the clinicians and the nurses, can pick up the role and assure the public that the vaccine is safe to all groups of people. We have not had adequate information for the public from the government encouraging this group of people to go for the vaccine and that it is safe … they need to declare that policy loud enough and then they [PLW] will come to us” (Policymaker, Nairobi, urban).

As seen with the policymakers, health workers described a lack of information and communication about the COVID-19 vaccine policy for pregnant and lactating women. This health worker from Nairobi recalled, “for this group [PLW] I believe, there is not so much information given, not so much about covid vaccine … so far I don’t think I have seen any guidelines on it” (Health worker, Nairobi, urban). Another health worker described needing more explicit information: “Pregnant, I am not yet sure. Until the time they tell us that it is good. They started saying that they can be given but you know we need to get it clear” (Health worker, Nairobi, urban). Others called for broader dissemination of the guidance on the use of vaccines in PLW, as seen with this health worker: “If it is not contraindicated let them get and let the message be passed. We get the message from the right people, maybe through the media, through the ones who are trained, that the vaccines can be given to pregnant mothers and it is safe, for breastfeeding mothers if it is also safe, let the information reach where it is supposed to reach” (Health worker, Garissa, rural).

PLW described how a lack of clear information made them question whether or not they should receive the vaccine: “And the question is concerning the new vaccine to protect us against Covid. My question is, is it advisable for an expectant mother to go for the vaccine because they have not come out clearly for us, so it is like we are in the dark, we don’t know whether we should go for it or we should wait. So we are somewhere in between” (Pregnant woman, Kakamega, rural). For those who were aware of the policy change, there was skepticism about the change: “They are saying right now pregnant women should be given covid-19 injection, why did they come all of a sudden and say women who are breastfeeding and pregnant women should be given the vaccination? … Back then, they were saying they should not be vaccinated … why did they come all of a sudden and say they should be injected?” (Lactating mother, Nairobi, urban).

### Vaccine safety concerns affecting policy for PLW

Across all participants, vaccine safety was reported as the major consideration for vaccine recommendations for PLW. Policymakers explained that the more restrictive policy for use in PLW was due to a lack of safety data, and several participants highlighted such evidence was required before a recommendation could be issued. Health workers expressed fears about adverse events if the vaccine were administered in pregnancy. PLW expressed the belief that their ineligibility was the result of unknown vaccine safety risks.

Policymakers stressed the importance of vaccine safety evidence for making a recommendation in pregnancy: “We just need to ensure that it is safe for them, once it is safe, we are free to vaccinate … We need to have a lot of research that it cannot cause organogenesis, it cannot cause abortion or preterm labor or placenta abruption, so it won’t bring so many issues” (Policymaker, Kakamega, urban). Another mentioned the requirement of safety evidence during lactation: “We need to have evidence on the safety profile for this vaccine on the pregnant woman herself, unborn baby and the safety profile on the breast milk … [If] there are no congenital effects for the unborn baby, there is no adverse effect on the baby who is breastfeeding then it’s given to the mother who is breastfeeding. That is the information that will be required” (Policymaker, Nairobi, urban). Another described how the risks were unknown, and that this led to a restrictive policy: “All the pregnant women and breastfeeding mothers were excluded from the vaccine because of the unknown risk of the exposure to the infants” (Policymaker, Nairobi, urban).

Questions about the safety of COVID-19 vaccines in pregnancy and lactation were echoed by many health workers: “Is there any chance if a lactating mother receives the vaccine does it have any effect on breastfeeding?” (Health worker, Kakamega, rural), and this health worker in Garissa: “Can pregnant mothers get the vaccine? Is it safe for them?” (Health worker, Garissa, rural). Some health workers expressed fear of harm to the fetus or newborn: “The information is that we do not have a guideline for them, for pregnant mothers, so we don’t know what the drug is going to do to the baby” (Health worker, Nairobi, urban); “I have heard it’s contraindicated [in pregnancy], the fetus is still young. In some cases, maybe it can cause some problems for the fetus” (Health worker, Kakamega, rural). Another health worker referred to vaccine safety research for a recommendation: “I believe the vaccine is safe but as earlier stated it’s still in trials. We are not sure about the safety with the pregnant mothers … or breastfeeding. So it’s something that is ongoing … I would recommend it so long as it is safe for use.” (Health worker, Kakamega, rural). One health worker indicated how they counseled pregnant women about safety as a reason why they were not receiving the vaccine: “What we do is counsel and tell them why. This is a new vaccine, research is still being done, they are trying to find out whether there are effects when you are given and you are expectant because you are not alone … So any effect that you will get will be transferred to the baby who is in utero so we want to prevent this … This being a new vaccine can it affect the baby as it grows? So we try to educate them and tell them why they are not getting” (Health worker, Nairobi, urban).

Many women perceived the exclusion of pregnant women as indicative of a safety risk: “I just heard that pregnant women are not being vaccinated, maybe because of the immunity and it is risky. I am not sure why but maybe they say that it is risky or something like that” (Pregnant woman, Garissa, rural). Similarly, a pregnant woman from Garissa expressed how the exclusion guided her decision. When asked if she would get the vaccine, she responded: “Personally no, just because of what I heard. Some people have this idea that this vaccination is still at the approval stage but it has not yet been approved completely. People are saying that it is in the trial stage so I won’t allow myself to try on something or risk myself to try something that I am not quite sure of” (Pregnant woman, Garissa, rural). However, some pregnant women expressed that data related to safety would motivate them: “If they say that for an expectant mother there is nothing wrong, I will go for it” (Pregnant woman, Kakamega, rural).

### Lack of prioritization of PLW related to national COVID-19 vaccine policy

Participants expressed concern over the lack of prioritization of PLW in the vaccination plans and the potential consequences. Some identified PLW as being at high risk for COVID-19 and felt this risk warranted the administration of vaccines in this population.

One policymaker felt the inclusion of PLW occurred too late and that pregnant women were largely forgotten: “Now until the policy came about 2 weeks ago there was ish-ish whether they are safe or not. But now they can immunize because the policy is that it is safe … You see, the pregnant were not part of the priority group anyway … the pregnant women and midwives have always been assumed, they have never been prioritized” (Policymaker, Nairobi, urban). Similarly, some policymakers expressed disappointment that PLW were initially excluded: “For me so long as you know the importance of that vaccine to the mother or to the client or to me myself, then you would go for that vaccination because it reduces the risks … That is why I was saying I wish they would have started with pregnant women but I read somewhere it needs more interrogations, more investigations … to ascertain the safety of these vaccines for pregnant women and lactating mothers … but I wish, if it was my wish, I wish they would have started with [PLW]” (Policymaker, Kakamega, urban).

Several health workers observed that PLW were at high risk for serious disease due to COVID-19 and expressed the need for protection: “If we get the guideline [for PLW], we would want mothers also to be vaccinated. Because now once this mother gets the COVID because of the lowered immunity those patients have, most of them are dying” (Health worker, Nairobi, urban). Another health worker from Kakamega felt PLW should not be turned away from vaccination: “I think they [health workers] should not turn them [PLW] away because these are people who are more at risk. So I think they should not turn them away” (Health worker, Kakamega, urban). Another health worker expressed PLW may feel ostracized due to ineligibility: “You know when they are turned away they usually feel ‘why are we being turned away and we are also at risk.’ In fact for their case they are at risk because their immunity is low. Yeah so they feel low, if there’s a vaccine that will accommodate all of them it will be better” (Health worker, Kakamega, urban). Another health worker similarly expressed that PLW may feel excluded due to the policy: “I don’t know the reason why we are being told it is ineligible for them (PLW) … if a mother went to receive the services and she is told she is not eligible am sure they may feel that they are left out” (Health worker, Garissa, rural).

## Discussion

Pregnant and lactating women, health workers, and policymakers described uncertainty of eligibility, suboptimal communication, vaccine safety concerns, and lack of prioritization of pregnant and lactating women in the national COVID-19 vaccine policy in Kenya. These findings have important implications for the uptake of vaccines among pregnant and lactating women, and can help inform future policy development and implementation for maternal immunization.

As data collection took place at a time when the policy for use of COVID-19 vaccines in pregnant and lactating women was changing, it was not surprising that participants provided mixed descriptions of the policy itself. Only a few policymakers described the more permissive policy stance, whereas health workers were largely unaware of the policy change, indicating suboptimal communication of the policy to implementers. Other policymakers and health workers reported no familiarity with any policy regarding COVID-19 vaccine use in pregnancy, indicating a broader policy implementation issue.

Kenya’s initial approach toward inclusion of pregnant and lactating women in COVID-19 vaccine deployment required an individual risk/benefit assessment by health care providers. Our interviews highlighted the difficulties in implementing this type of policy, as none of our health workers described having sufficient information about the vaccines to conduct such assessments. These types of individual risk assessment policy recommendations for pregnant and lactating women were common at the outset of the COVID-19 vaccine roll-out,^4^ which may have hindered access and implementation. Kenya’s current policy, which categorizes pregnant individuals as high-risk, provides clear guidance to health care providers. Our participants reported an understanding of the eligibility and prioritization policies for other groups, signifying that effective informational channels exist for national policy dissemination and could be leveraged for communication of clearly articulated policies and guidelines for vaccination of pregnant and lactating women.

Unclear policy guidance posed challenges for health workers including inadequate counseling and resulting in health workers turning away pregnant and lactating women. For pregnant and lactating women, their perception of ineligibility indicated vaccine safety risks during pregnancy and/or lactation, and this perception can lead to increased vaccine hesitancy and lower vaccine uptake. Our results are in line with the available evidence related to vaccine intentions of pregnant and lactating women globally,^28^ and in Kenya. A multi-site study that examined vaccine intentions among pregnant women found that 41% of the pregnant women in the study from Kenya reported they would not get a COVID-19 vaccine, primarily due to questions about safety (36%) and fear of adverse events (30%).^29^ Health care providers are consistently cited as a trusted source of information for vaccination,^30–32^ and should therefore be equipped with information as to why pregnant and lactating women may or may not be eligible for a vaccine product. The development and dissemination of guidelines for vaccine counseling would help health workers navigate conversations with patients and mitigate vaccine hesitancy.^33^

The lack of vaccine safety data was repeatedly cited by study participants as the reason pregnant and lactating women were initially ineligible to receive COVID-19 vaccines in Kenya. Policymakers and health workers reported they would recommend the vaccine to pregnant and lactating women only after there was adequate evidence indicating that COVID-19 vaccines were safe for use in pregnancy, followed by government guidelines. Given that policymakers and health workers rely heavily on vaccine safety evidence to make recommendations,^34^ it is imperative that future vaccine clinical trials include pregnant and lactating individuals to generate the evidence required for maternal immunization policy at the outset of vaccine introduction. Additionally, our findings indicated that the position statement from the Kenya Obstetrical and Gynecological Society appeared to have influenced the change in the national position, highlighting how professional societies have a role to play in advocating for maternal health and immunization policy.

The risk of COVID-19 in pregnancy was also raised by policymakers and health workers as an important indication for vaccine use in pregnancy. The lack of prioritization of pregnant and lactating women in vaccine implementation concerned many respondents who felt this high-risk group was being left behind. Unfortunately, there are no national estimates of the burden of COVID-19 in pregnant people in Kenya, but one study found a greater incidence of SARS-CoV-2 infection among pregnant antenatal care patients compared to postpartum mothers.^35^ In Kenya, there are an estimated 2,380,000 pregnancies per year, representing a large population that could be disproportionately impacted by COVID-19 and benefit from vaccine prioritization.^36^ The collection and dissemination of data at country and subnational levels would help elucidate the COVID-19 burden among pregnant women in Kenya, highlight vaccine access gaps, and tailor vaccine policies, communication, and operational plans to address inequities in access.

This study is not without limitations. As described previously, this study was conducted during a dynamic policy environment in Kenya, resulting in data that was collected before and after a new policy on vaccine use in pregnancy and lactation was announced. However, this allowed us to examine in part how participants responded to this guidance change and to evaluate its dissemination. This study has several strengths. We used a holistic approach to understand the policy, as we interviewed pregnant and lactating women, health workers, and policymakers from diverse communities. Due to the qualitative nature of our study, we were able to identify factors perceived as influencing vaccine policy and subsequent uptake. Our study is one of the first to examine COVID-19 vaccine policies related to pregnant and lactating women, and our findings can uniquely inform how future maternal immunization policy is introduced and implemented.

This study demonstrated how COVID-19 vaccine policy played a central role in influencing vaccine behaviors among pregnant and lactating women in Kenya. Our findings highlight the need for more effective communication of COVID-19 vaccine policy targeting policy makers and health care workers, and the importance of prioritization of pregnant and lactating women in COVID-19 vaccination policy and campaigns. To prevent future inequities in vaccine access for pregnant and lactating women, it is critical that pregnant and lactating women are included in vaccine research and that data on vaccine uptake among pregnant and lactating women are collected and disseminated.
